# Toxic Leadership and Employee Outcomes Among Mental Health Nurses: Examining the Associations with Psychological Distress, Somatic Symptoms, and Organizational Citizenship Behavior Through Structural Equation Modeling

**DOI:** 10.3390/healthcare14152258

**Published:** 2026-07-24

**Authors:** Manal Saleh Moustafa Saleh, Hamad G. Dailah, Enas Mohamed Lotfy, Mohammed A. Majrabi, Saud Baraki Almutairi, Shabab Mohammed Alghamdi, Abdulrahman Mahdali Aljubairi, Mishal Khaled Alholibeh, Safaa Gaber Aly Salem, Riham Hashem Fathi

**Affiliations:** 1 Department of Nursing Sciences, College of Applied Medical Science, Shaqra University, Shaqra 11961, Saudi Arabia; 2 Nursing Administration, Faculty of Nursing, Zagazig University, Zagazig 44519, Egypt; 3 College of Nursing and Health Sciences, Jazan University, Jazan 82817, Saudi Arabia; hdaelh@jazanu.edu.sa; 4 Public Health Nursing Department, College of Nursing, Northern Border University, Arar 73213, Saudi Arabia; enas.mahmoud@nbu.edu.sa; 5 Eradah & Mental Health Hospital, Jazan Health Cluste, Jazan 45142, Saudi Arabia; mmajrab@outlook.sa; 6 Anesthesia Department, King Fahad Armed Forces Hospital, Ministry of Defense, Riyadh 11159, Saudi Arabia; 7 Anesthesia Department, Prince Sultan Military Medical City, Ministry of Defense, Riyadh 12233, Saudi Arabia; 8 Department of Maternity, Obst & Gyn Nursing, College of Nursing, Princess Nourah bint Abdulrahman University, P.O. Box 84428, Riyadh 11671, Saudi Arabia; 9 Faculty of Medicine, Zagazig University, Zagazig 44519, Egypt

**Keywords:** motivation, toxic leadership, psychological distress, somatic symptoms, mental health nursing, organizational citizenship behavior

## Abstract

**Highlights:**

**What are the main findings?**

- Toxic leadership was significantly associated with psychological distress among mental health nurses.
- Psychological distress was strongly associated with somatic symptoms.
- Intrinsic motivation was positively associated with organizational citizenship behavior.
- The hypothesized mediating role of intrinsic motivation was not supported.
- No significant direct association was found between toxic leadership and organizational citizenship behavior.

**What are the implications of the main findings?**

- Healthcare organizations should implement strategies to reduce toxic leadership behaviors in clinical settings.
- Leadership development and supportive management practices may improve nurses’ psychological well-being.
- Enhancing intrinsic motivation can strengthen positive workplace behaviors and organizational performance.
- Mental health support programs may help reduce psychological distress and somatic symptoms among nurses.
- Promoting healthy work environments may improve quality of care and patient outcomes in mental health settings.

**Abstract:**

**Background:** Mental health nurses work in high-pressure environments where leadership styles strongly influence well-being and job performance. Toxic leadership, including abusive and authoritarian behaviors, can contribute to psychological distress, somatic symptoms, reduced job satisfaction, and lower organizational effectiveness. It may also decrease Organizational Citizenship Behavior (OCB), which reflects voluntary positive workplace behaviors. Intrinsic motivation may help buffer these negative effects by enhancing employees’ internal drive and resilience. Therefore, this study examines the associations between toxic leadership, psychological distress, somatic symptoms, and organizational citizenship behavior among mental health nurses while exploring the potential role of intrinsic motivation. **Methods:** This descriptive-correlational study, based on Structural Equation Modeling (SEM), was conducted at the Psychiatric Inpatient Department of Mansoura University Hospital. A total of 117 mental health nurses were recruited through simple random samplings. Data were collected using four standardized tools: the Toxic Leadership Scale, the Brief Symptom Inventory (BSI), the Intrinsic Motivation Inventory (IMI), and the Organizational Citizenship Behavior (OCB) Scale, starting from the end of May and completed by the end of June 2024. Data analysis was conducted using IBM SPSS 22 and AMOS 22, employing descriptive statistics, Pearson’s correlations, independent t-tests, and Structural Equation Modeling (SEM). **Results:** Toxic leadership was positively associated with psychological distress (β = 0.46, *p* < 0.001). Psychological distress was strongly associated with somatic symptoms (β = 0.99, *p* < 0.001). Intrinsic motivation was positively associated with organizational citizenship behavior (β = 0.31, *p* < 0.001). However, intrinsic motivation was not significantly associated with psychological distress or somatic symptoms, and no significant direct association was observed between toxic leadership and organizational citizenship behavior. **Conclusions:** Toxic leadership was significantly associated with higher levels of psychological distress among mental health nurses, while psychological distress was strongly associated with somatic symptoms. Intrinsic motivation was positively associated with organizational citizenship behavior. However, the hypothesized mediating role of intrinsic motivation was not supported by the structural model. These findings highlight the importance of addressing toxic leadership and promoting supportive workplace environments to enhance nurses’ well-being and organizational outcomes. **Relevance for Clinical Practice:** Addressing toxic leadership is essential for enhancing nurses’ well-being and organizational outcomes. Interventions targeting leadership behaviors and promoting supportive workplace environments may help reduce psychological distress and its associated somatic symptoms among mental health nurses. In addition, creating workplace conditions that support intrinsic motivation may encourage organizational citizenship behavior and professional engagement, ultimately benefiting clinical practice and patient care quality.

## 1. Introduction

The role of mental health nurses is critical within healthcare systems, given their responsibility for delivering complex and often emotionally demanding care to patients with mental health conditions [1]. Leadership plays an important role in workplace environments, and has been associated with employees’ performance as well as their mental and physical well-being [2]. In the healthcare sector, particularly among mental health nurses, leadership styles can be significantly associated with stress levels, job satisfaction, and behaviors that extend beyond formal duties, such as organizational citizenship behavior (OCB) [3]. However, the presence of toxic leadership in healthcare environments is increasingly recognized as a factor associated with poorer well-being and performance among healthcare professionals. Mental health nurses, who face intense job demands, are particularly vulnerable to the negative effects of toxic leadership, which can manifest in psychological distress and somatic symptoms [4].

### 1.1. Association Between Toxic Leadership and Mental Health: Nurses’ Psychological Distress and Somatic Symptoms

Toxic leadership, characterized by behaviors such as abuse of power, manipulation, and a lack of empathy, creates a hostile work environment with significant implications for mental health nurses’ psychological and physical health, as well as their organizational behaviors [5]. Studies have shown that toxic leadership is associated with increased levels of stress, depression, and burnout among employees, particularly within high-stress professions like nursing [6,7]. For mental health nurses, the impact of toxic leadership may be even more pronounced due to the emotional labor and patient advocacy roles they assume [8].

Psychological distress and somatic symptoms, such as fatigue and musculoskeletal pain, are commonly reported among nurses working under toxic leadership conditions [9]. Toxic leaders often undermine autonomy and foster a climate of fear, which exacerbates feelings of helplessness and low morale among nurses and may be associated with poorer mental and physical health [10]. Research has shown that toxic leadership significantly contributes to increased stress, emotional exhaustion, and depressive symptoms among healthcare workers [11]. In the context of mental health nursing, such leadership exacerbates job-related stress. It negatively impacts nurses’ psychological and physical health, leading to somatic symptoms such as fatigue and sleep disturbances [12,13].

Mental health nurses often operate in high-stress environments that demand emotional labor, making them particularly susceptible to burnout, depression, and physical health issues. Toxic leadership undermines employees’ ability to cope with stress [14]. It creates a hostile work environment, contributing to mental and physical strain and affecting nurses’ overall well-being. Therefore, it is important to analyze the leader’s behavior in context to deal effectively with toxic leaders and understand their behavior [15].

### 1.2. Organizational Citizenship Behavior

Organizational Citizenship Behavior (OCB) refers to voluntary, extra-role activities that employees undertake to support the functioning of their organization. OCB is particularly beneficial in healthcare, where collaboration and teamwork are essential to patient care [16]. Research in nursing contexts has shown that positive leadership has been associated with higher levels of OCB by promoting job satisfaction and motivation. Conversely, toxic leadership has been associated with lower OCB, as employees feel disengaged and less motivated to contribute beyond their assigned tasks [17,18].

The presence of toxic leadership often discourages proactive engagement and cooperation, further reducing OCB among employees [19]. Intrinsic motivation, defined as an individual’s internal drive to engage in tasks for personal satisfaction and a sense of achievement, has been proposed as a potential mediator between the negative effects of toxic leadership and OCB [20]. Nurses with high intrinsic motivation are more likely to maintain OCB even under challenging conditions, driven by their commitment to patient care and professional values [21].

### 1.3. Intrinsic Motivation as a Mediator

Intrinsic motivation is the drive to engage in activities for their inherent satisfaction rather than external rewards [22]. In healthcare, intrinsic motivation plays a crucial role in maintaining job performance and engagement, particularly in challenging environments. Toxic leadership, however, undermines intrinsic motivation by fostering an environment that stifles autonomy and discourages personal growth [23].

Self-Determination Theory (SDT) provides a useful theoretical framework for understanding how workplace environments influence employee motivation, well-being, and work-related behaviors. According to SDT, individuals function optimally when three basic psychological needs, autonomy, competence, and relatedness, are supported within their work environment [24,25]. Leadership behaviors play a critical role in shaping these psychological needs. Supportive leadership may foster intrinsic motivation by promoting autonomy and professional growth, whereas toxic leadership may undermine employees’ sense of competence, relatedness, and psychological safety.

Within this framework, intrinsic motivation was proposed as a potential mechanism linking toxic leadership to employee outcomes. Nurses with higher levels of intrinsic motivation may rely on internal sources of satisfaction, professional commitment, and personal meaning when facing workplace stressors, thereby helping to maintain positive work behaviors and psychological well-being [26]. Previous studies have suggested that intrinsic motivation is associated with greater resilience, lower burnout, and stronger organizational citizenship behaviors among healthcare professionals [27,28]. Therefore, SDT provides a theoretical basis for examining the relationships among toxic leadership, intrinsic motivation, psychological distress, somatic symptoms, and organizational citizenship behavior in mental health nurses.

Understanding intrinsic motivation as a protective factor thus provides valuable insight into how mental health nurses might maintain professional engagement and cope more effectively with toxic leadership [29]. This research seeks to offer practical insights into supporting the mental health of nurses, enhancing organizational outcomes, and fostering healthier work environments within the healthcare sector. Therefore, this study aims to examine the associations between toxic leadership and mental health nurses’ depression, somatic symptoms, and OCB, with a specific focus on the mediating role of intrinsic motivation, by exploring how intrinsic motivation influences the relationships between depression, somatic symptoms, and OCB.

Although previous studies have examined the associations between toxic leadership and employee well-being, limited evidence is available regarding these relationships among mental health nurses, particularly within psychiatric inpatient settings in Middle Eastern healthcare contexts. Furthermore, the potential role of intrinsic motivation in explaining the relationships between toxic leadership, psychological distress, somatic symptoms, and organizational citizenship behavior remains insufficiently understood. Addressing this gap may contribute to a better understanding of occupational health risks and organizational outcomes among mental health nursing staff.

### 1.4. Hypotheses

1. Toxic Leadership and Well-Being Outcomes

**H1a:**  *Toxic leadership is positively associated with psychological distress among mental health nurses*.

**H1b:**  *Toxic leadership is positively associated with somatic symptoms among mental health nurses*.

2. Intrinsic Motivation

**H2a:**  *Intrinsic motivation mediates the relationship between toxic leadership and psychological distress among mental health nurses*.

**H2b:**  *Intrinsic motivation mediates the relationship between toxic leadership and somatic symptoms among mental health nurses*.

**H2c:**  *Intrinsic motivation is positively associated with organizational citizenship behavior among mental health nurses*.

**H2d:**  *Higher levels of intrinsic motivation are associated with lower psychological distress among mental health nurses*.

**H2e:**  *Higher levels of intrinsic motivation are associated with lower somatic symptoms among mental health nurses*.

3. Organizational Citizenship Behavior

**H3a:**  *Toxic leadership is negatively associated with organizational citizenship behavior among mental health nurses*.

**Figure 1:**
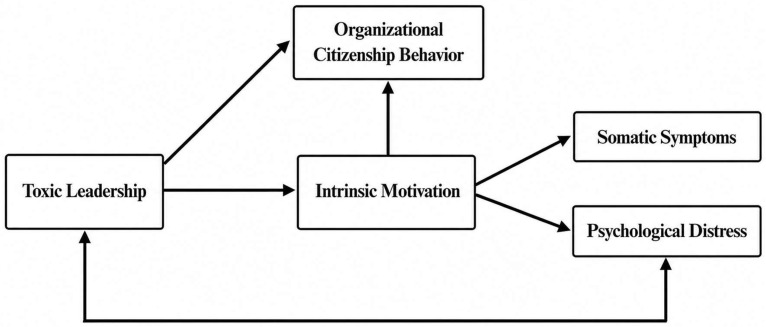
Conceptual model illustrating the hypothesized associations between toxic leadership, psychological distress, somatic symptoms, intrinsic motivation, and organizational citizenship behavior.

Considering the hypotheses mentioned above, the conceptual research model is presented in Figure 1.

### 1.5. The Study Objective

The objective of the study is to examine the associations between toxic leadership, psychological distress, somatic symptoms, and organizational citizenship behavior among mental health nurses while exploring the potential role of intrinsic motivation.

## 2. Methodology

### 2.1. Study Design

A descriptive-correlational design based on Structural Equation Modeling (SEM) was chosen for this study.

### 2.2. Study Setting

The study was conducted at the Psychiatric Inpatient Department in Mansoura University Hospital. This hospital is in the city of El Mansoura, the capital of the Dakahlia governorate in the Delta region. The inpatient department provides psychiatric management for adults and children with psychotic, neurotic, or addictive disturbances. All clinical services are provided free of charge for most patients.

### 2.3. Participants

Participants were selected using a simple random sampling method. Eligible participants were registered mental health nurses working in psychiatric units at the Mansoura University Hospital during the data collection period. Nurses who had at least one year of clinical experience and who were willing to participate were included in the study. Nurses on extended leave during the data collection period or those who declined participation were excluded. A list of eligible nurses was obtained from the nursing administration, and participants were selected using a simple random sampling procedure. Participation was entirely voluntary, and informed consent was obtained before data collection.

An open-source sample size calculator (https://www.calculator.net/sample-size-calculator.html, accessed on 1 May 2024) was utilized to determine the required sample size. The calculation was based on the total population of 167 nurses at the Mansoura University Hospital, using a confidence interval level of 95%, a margin of error of 5.0%, and a population proportion of 50%. This resulted in a required sample size of 117 mental health nurses. Both male and female nurses who voluntarily expressed interest in participating were included. The simple random sampling process continued until the desired sample size was achieved. The sample size is within the acceptable range for applying Structural Equation Modeling (SEM) using AMOS; our study sample size (N = 117) is supported by previous research by Wolf et al. [30] and Hair et al. [31]. The study focused on a specialized and relatively homogeneous group of mental health nurses working in psychiatric settings. Although recruiting large samples from this population can be challenging, the professional specificity of the participants enhances the contextual relevance of the findings.

### 2.4. Instruments Used in the Study

The data for this study were collected using four standardized scales that were originally developed in English and implemented in Arabic.

#### 2.4.1. The Toxic Leadership Scale

Toxic leadership was assessed using the 15-item questionnaire developed by Schmidt [7]. The scale measures five dimensions, each represented by three items: abusive supervision (AS), authoritarian leadership (AL), narcissism (N), self-promotion (SP), and unpredictability (U). All items begin with the phrase “My current supervisor”, and example items for each dimension include: “Holds subordinates responsible for things outside their job descriptions” (abusive supervision), “Controls how subordinates complete their tasks” (authoritarian leadership), “Has a sense of personal entitlement” (narcissism), “Drastically changes his/her demeanor when his/her supervisor is present” (self-promotion), and “Allows his/her current mood to define the climate of the workplace” (unpredictability). The internal consistency (coefficient alpha) values for the dimensions were as follows: abusive supervision (α = 0.79), authoritarian leadership (α = 0.84), narcissism (α = 0.81), self-promotion (α = 0.85), and unpredictability (α = 0.85).

##### Scoring System

All items were rated on a 5-point Likert scale, with responses ranging from 1 (“Strongly Disagree”) to 5 (“Strongly Agree”). The Toxic Leadership Scale (TLS) measures perceived toxic leadership behaviors, with scores ranging from 15 to 75. The total score can be categorized into three levels: 

Low Toxic Leadership (15–34; less than 50%): This range indicates a minimal perception of toxic leadership in the workplace. 

Moderate Toxic Leadership (35–54; 50% to less than 75%): Scores in this range suggest the presence of some toxic leadership traits.

High Toxic Leadership (55–75; 75% to 100%): A score in this range reflects a severe presence of toxic leadership [7].

#### 2.4.2. The Brief Symptom Inventory: Psychological Distress and Somatic Symptoms

The self-reported Brief Symptom Inventory (BSI) by Derogatis [32] was used to measure participants’ psychological distress and somatic symptoms during the past week, including the day they completed the inventory. Example items included “To what extent did you suffer from no interest in things?” (psychological distress) and “To what extent did you suffer from pain in the chest or heart?” (somatic symptoms). The questionnaire consisted of 53 items in total.

##### Scoring System

Responses were recorded on a 5-point Likert scale, ranging from “not at all = 0” to “extremely = 4”. The total scores were obtained by summing the responses across all 53 items and were categorized into three levels: 

Low Symptom Severity (0–105; less than 50%): Minimal presence of psychological distress and somatic symptoms.

Moderate Symptom Severity (106–158; 50% to less than 75%): Presence of noticeable psychological distress and somatic symptoms.

High Symptom Severity (159–212; 75% to 100%): Severe psychological distress and somatic symptoms, indicating significant distress [32].

#### 2.4.3. The Intrinsic Motivation Inventory (IMI)

The Intrinsic Motivation Inventory (IMI), in its validated short form developed and psychometrically tested by Ostrow and Heffernan [33], is a widely used self-report instrument for measuring intrinsic motivation and self-determination. In the present study, the instrument was used in its original validated form without any modification. The scale is particularly relevant for understanding how motivation influences behavior and performance. The IMI includes 19 items divided into several subscales. Interest/Enjoyment reflects the inherent pleasure or satisfaction derived from a task. For instance, items like “This assignment was fun to do” emphasize enjoyment as a primary indicator of intrinsic motivation. Competence assesses individuals’ perceptions of their abilities in completing a task. Statements such as “I am satisfied with my performance on this assignment” highlight the role of self-efficacy. Autonomy measures the sense of volition or choice in performing an activity. Reverse-coded items like “I did this assignment because I had to” evaluate whether participants feel constrained or autonomous. Belonging captures the sense of social connection and interaction, particularly relevant in collaborative environments. Items such as “My colleagues and I could likely become friends if we interacted a lot” assess this dimension.

##### Scoring System

Responses were recorded using a 5-point Likert scale, ranging from 1 (Strongly Disagree) to 5 (Strongly Agree). The total scores were obtained by summing the responses across all 19 items, which were then categorized into three levels: 

Low Intrinsic Motivation (19–47; less than 50%): Indicates a minimal level of intrinsic motivation. 

Moderate Intrinsic Motivation (48–71; 50% to less than 75%): Suggests a moderate level of intrinsic motivation. 

High Intrinsic Motivation (72–95; 75% to 100%): Reflects a strong level of intrinsic motivation [33].

#### 2.4.4. The Organizational Citizenship Behavior (OCB) Scale

Kumar and Shah [34] developed the Organizational Citizenship Behavior (OCB) Scale, which was adapted to evaluate organizational citizenship behavior among nurses. This scale includes 15 items divided into five distinct domains: altruism, courtesy, civic virtue, sportsmanship, and conscientiousness. Each domain is represented by three items designed to measure specific aspects of OCB. The altruism domain focuses on behaviors demonstrating a willingness to help others, such as “I am always ready to lend a helping hand to those around me”. The courtesy domain assesses efforts to maintain positive relationships and prevent conflict, with items like “I always try to avoid creating problems for co-workers”.

The civic virtue domain evaluates engagement with and awareness of organizational matters, exemplified by the item “I keep myself updated with organizational announcements and memos”. The sportsmanship domain measures an individual’s ability to handle challenges without complaints, as seen in the statement “I always require frequent doses of motivation to get the work done”. Finally, the conscientiousness domain reflects self-discipline and adherence to workplace norms, illustrated by the item “I do not take extra or long breaks while on duty”.

##### Scoring System

The responses to the 15 items were recorded using a 5-point Likert scale, ranging from 1 (strongly disagree) to 5 (strongly agree). This scoring system allows for a nuanced assessment of OCB across the five domains. The total scores were obtained by summing the responses across all 15 items and grouping them into three levels: 

Low OCB (15–37; less than 50%): Indicates a minimal level of organizational citizenship behavior. 

Moderate OCB (38–56; 50% to less than 75%). 

High OCB (57–75; 75% to 100%): Reflects a strong level of organizational citizenship behavior where individuals consistently engage in supportive and responsible workplace behaviors [34].

#### 2.4.5. Demographic Data of Study Participants

The participants’ demographic information was obtained, including their age, gender, marital status, years of experience, and department.

### 2.5. Translation Procedures, Pilot Study, and the Tools’ Validity and Reliability

To ensure full comprehension of the study measures among Arabic-speaking nurses, all instruments were translated into Arabic. A panel of five bilingual university professors (English and Arabic) reviewed the translation, followed by a back-translation process conducted by another panel of five university professors. Additionally, ten nursing professors assessed the content and face validity of the translated measures. Their evaluation confirmed the validity of the instruments, with no modifications required.

A pilot study was conducted with 11 mental health nurses (10% of the sample) to assess the linguistic accuracy, clarity, and cultural appropriateness of the translated scales. Despite being well-established in English, the instruments required contextual adaptation for Arabic-speaking participants. The pilot’s study ensured that all items were comprehensible and relevant before full-scale data collection.

Reliability analysis was conducted using the test–retest method over a two-week interval, followed by Cronbach’s alpha reliability testing using SPSS. The results demonstrated high internal consistency across all scales. The Toxic Leadership Scale: Overall α = 0.974, with subscale reliability ranging from 0.933 to 0.971. Psychological distress and Somatic Symptoms Scale: α = 0.944. The Intrinsic Motivation Inventory (IMI): α = 0.927. The Organizational Citizenship Behavior (OCB) Scale: α = 0.981. These findings confirm the high reliability and validity of the study instruments, ensuring their suitability for assessing the targeted psychological and behavioral constructs.

### 2.6. Overcoming the Problem of Common Method Biases

To minimize common method bias (CMB), several procedural, design, and statistical strategies were employed. Participant confidentiality and anonymity were ensured to reduce social desirability bias and encourage honest responses. The questionnaire was carefully structured to separate different constructs, incorporate varied response formats, and provide clear instructions to minimize response patterning. A pilot study involving 10% of the target sample was conducted to improve item clarity and eliminate potential ambiguities. In addition, Harman’s single-factor test was performed to statistically assess the presence of common method bias. The first unrotated factor accounted for less than 50% of the total variance, indicating that common method bias was unlikely to have substantially influenced the study findings. Collectively, these procedural and statistical measures support the robustness and validity of the data collected.

### 2.7. Data Collection

Self-reported assessments from mental health nurses were employed to collect data for this study, starting from the end of May, and were completed by the end of June 2024. Before any data were collected, the necessary ethics approvals were acquired. The study’s objectives were conveyed to the nurses during their initial encounter, which took place either via Google Meet or at each hospital. The lead investigator also requested their aid in facilitating the data collection process. As a result, nurses had higher perceived anonymity and lower possible response bias.

### 2.8. Ethical Considerations and Consent to Contribute

This study was conducted following the ethical principles outlined in the Declaration of Helsinki (DoH Oct 2008). The Ethics Committee of the Faculty of Nursing, Zagazig University, Egypt, granted ethical approval (reference number 188-27 in 6 May 2024). All necessary information about the study was introduced in the first section of the sheet. The questionnaire included a statement related to the aim and nature of the study. The respondents were guaranteed the privacy and confidentiality of their answers, the voluntary nature of their involvement, and that their absence would not result in any negative outcomes. Participants gave their informed consent in accordance with the criteria outlined in the Declaration of Helsinki. Participants had the right to withdraw from the study at any time.

### 2.9. Statistical Analysis

Data analysis was conducted using IBM SPSS Statistics (Version 22) and IBM SPSS AMOS (Version 22). Descriptive statistics, including frequencies and percentages, were used to summarize the demographic characteristics of the participants, while means and standard deviations were calculated to assess the three primary study variables: toxic leadership, psychological distress, and organizational citizenship behavior (OCB). To examine differences in study variables based on demographic characteristics, independent sample t-tests were performed. The relationships among the key research variables were assessed using Pearson’s correlation analysis.

A structural equation model (SEM) was employed to examine the hypothesized relationships among the study variables and to assess both direct and indirect effects within the proposed conceptual framework. The potential mediating role of intrinsic motivation in the relationships between toxic leadership and organizational citizenship behavior, psychological distress, and somatic symptoms was evaluated. The significance of the indirect effects was assessed using the Sobel test. The mediation effect was considered statistically significant when the absolute Sobel Z-value exceeded 1.96 at the 0.05 significance level. The Sobel test was applied to the following indirect pathways: Toxic Leadership → Intrinsic Motivation → Organizational Citizenship Behavior; Toxic Leadership → Intrinsic Motivation → Psychological Distress; and Toxic Leadership → Intrinsic Motivation → Somatic Symptoms. The Sobel test statistic was calculated using the following formula:

$$Z\;value\;=\;\frac{ab}{\sqrt{b^{2}\;{S_{a}}^{2}\;+\;a^{2}\;{S_{b}}^{2}}}$$

where *a* is the regression coefficient of the independent variable on the mediator variable, *b* is the regression coefficient of the mediator variable on the dependent variable, *S_a_* is the standard error of the relationship between the independent variable and the mediator variable, and *S_b_* is the standard error of the relationship between the mediator variable and the dependent variable. To ensure the reliability and validity of the study instruments, Cronbach’s alpha was calculated to validate the measurement model and confirm the distinctiveness of the study constructs, and statistical significance was determined at a two-tailed *p*-value of <0.05.

Model fit was evaluated using multiple goodness-of-fit indices, including χ^2^/df, the Comparative Fit Index (CFI), Tucker–Lewis Index (TLI), Root Mean Square Error of Approximation (RMSEA), and Standardized Root Mean Square Residual (SRMR). Acceptable model fit was defined as CFI and TLI ≥ 0.90, RMSEA ≤ 0.08, and SRMR ≤ 0.08.

Structural model evaluation was based on the significance of path coefficients and the overall theoretical consistency of the proposed model. Standardized regression coefficients (β), standard errors, critical ratios, and p-values were used to assess the hypothesized relationships among study variables.

## 3. Results

Table 1 presents the demographic characteristics of the participating mental health nurses and differences in the study variables across demographic groups. Most participants were aged 30–35 years (40.2%), female (73.5%), and single (53.0%). The majority had less than five years of experience (89.7%) and worked in mental health departments (84.6%). No statistically significant differences were observed in most study variables according to gender, marital status, department, or years of experience (*p* > 0.05). However, significant differences were identified across age groups for toxic leadership and intrinsic motivation (*p* < 0.05), indicating that perceptions of these variables varied according to participants’ age.

Table 2 presents the correlations among the study variables. Toxic leadership was positively correlated with intrinsic motivation (r = 0.276, *p* < 0.01), psychological distress (r = 0.454, *p* < 0.01), and somatic symptoms (r = 0.427, *p* < 0.01). Intrinsic motivation was positively associated with organizational citizenship behavior (r = 0.453, *p* < 0.01), psychological distress (r = 0.279, *p* < 0.01), and somatic symptoms (r = 0.278, *p* < 0.01). A strong positive correlation was observed between psychological distress and somatic symptoms (r = 0.947, *p* < 0.01). No significant correlations were found between toxic leadership and organizational citizenship behavior or between organizational citizenship behavior and either psychological distress or somatic symptoms.

Table 3 presents the direct and indirect effects among the study variables estimated through the structural model. Toxic leadership demonstrated a significant positive direct association with psychological distress (β = 0.46, *p* < 0.001). Intrinsic motivation was positively associated with organizational citizenship behavior (β = 0.31, *p* < 0.001), whereas psychological distress showed a strong positive association with somatic symptoms (β = 0.99, *p* < 0.001). No significant direct associations were observed between toxic leadership and intrinsic motivation, toxic leadership and organizational citizenship behavior, or intrinsic motivation and psychological distress or somatic symptoms.

Correspondingly, a significant indirect association was observed between toxic leadership and somatic symptoms (β = 0.460, *p* = 0.004, 95% CI = 0.265–0.684). This finding indicates that the structural model identified a statistically significant indirect association between toxic leadership and somatic symptoms. However, because the structural paths involving intrinsic motivation were not statistically significant, this indirect association does not support the hypothesized mediating role of intrinsic motivation. In addition, no significant indirect effect was found between toxic leadership and organizational citizenship behavior (β = −0.036, *p* = 0.227).

**Figure 2:**
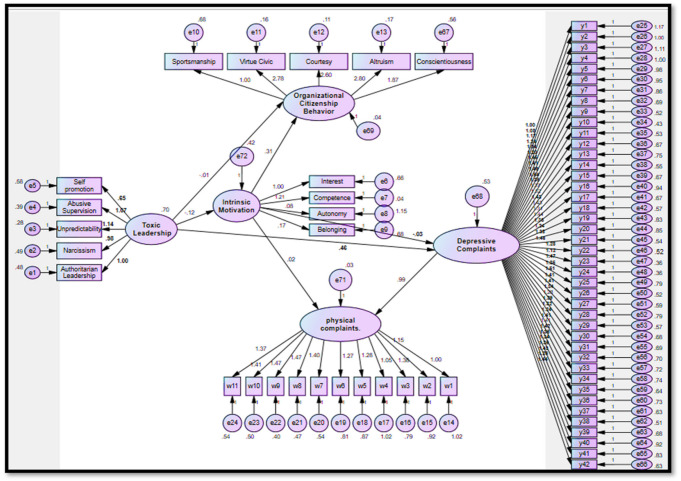
Structural model illustrating the relationships among toxic leadership, intrinsic motivation, psychological distress, and somatic symptoms among mental health nurses (n = 117). Note: Values displayed on the paths represent standardized regression coefficients (β). Error terms are indicated by (e). Note: Depressive Complaints and Physical Complaints in the figures correspond to Psychological Distress and Somatic Symptoms, respectively, as referred to throughout the manuscript.

**Figure 3:**
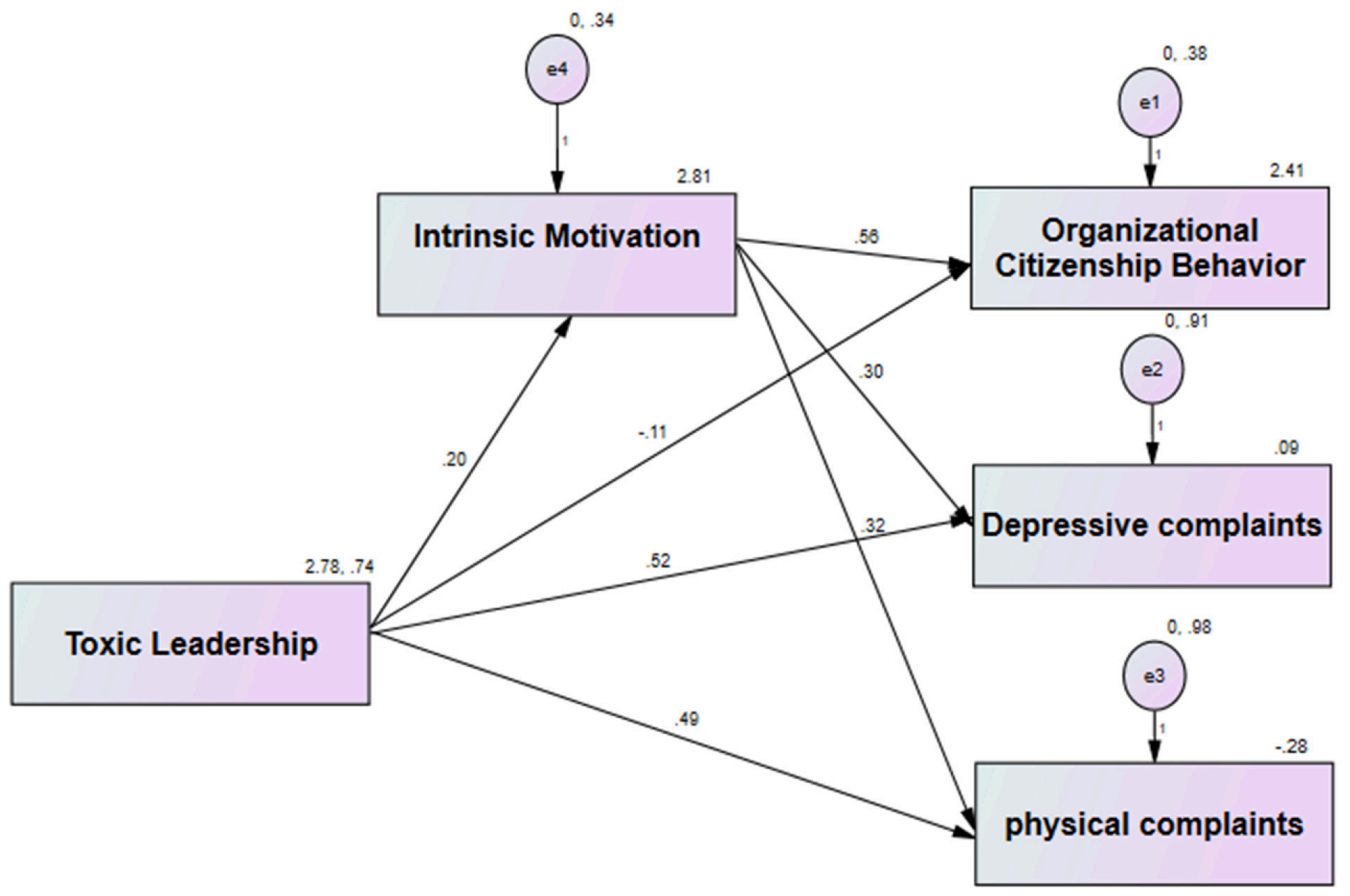
Structural path model showing the direct and indirect associations among toxic leadership, intrinsic motivation, organizational citizenship behavior, psychological distress, and somatic symptoms (n = 117). Note: The values above the path lines (straight lines) show the path coefficients of the independent variables under study, while the values (e) represent the path coefficients corresponding to the error. Note: Depressive Complaints and Physical Complaints in the figures correspond to Psychological Distress and Somatic Symptoms, respectively, as referred to throughout the manuscript.

Figure 2 and Figure 3 illustrate the structural models, which indicate that toxic leadership was significantly associated with higher levels of psychological distress among mental health nurses. However, toxic leadership was not significantly associated with intrinsic motivation or organizational citizenship behavior. Furthermore, intrinsic motivation was significantly associated with organizational citizenship behavior but showed no significant association with psychological distress or somatic symptoms.

Psychological distress demonstrated a strong positive association with somatic symptoms, indicating that nurses reporting higher levels of psychological distress were also more likely to report increased somatic symptoms. In addition, toxic leadership exhibited a significant indirect association with somatic symptoms through an indirect pathway estimated in the structural model.

The indices in Table 4 show that the proposed structural model provided a good fit to the observed data. The χ^2^/df ratio was 3.491, which falls within the acceptable range. In addition, the RMSEA value was 0.047, indicating a close model fit. The incremental fit indices also exceeded the recommended threshold of 0.90, including NFI (0.98), RFI (0.97), AGFI (0.92), and TLI (0.97). Collectively, these results support the adequacy of the structural model for examining the hypothesized relationships among the study variables.

## 4. Discussion

Toxic leadership is increasingly prevalent in today’s organizations, including healthcare organizations [35]. The present study examined the relationships among toxic leadership, intrinsic motivation, organizational citizenship behavior, psychological distress, and somatic symptoms among mental health nurses. The structural model demonstrated a good fit to the data, supporting the suitability of the proposed framework. The findings revealed that toxic leadership was significantly associated with psychological distress, while intrinsic motivation was positively associated with organizational citizenship behavior. Furthermore, psychological distress showed a strong positive association with somatic symptoms among mental health nurses.

A strong positive correlation was observed between psychological distress and somatic symptoms, indicating that as mental health issues like psychological distress increase, physical health problems also escalate. This result is consistent with the biopsychosocial model, which holds that because psychological and physiological processes are interrelated, mental health problems frequently manifest physically [36]. According to studies, somatic complaints such as pain, exhaustion, and gastrointestinal problems are commonly reported by people who are depressed [37]. Also, this finding echoes the work of Kim & Jeong [38] who identified similar associations in healthcare workers, where higher levels of stress and depression were linked to an increase in musculoskeletal disorders, fatigue, and other physical ailments. This aligns with the theory that chronic stress and emotional distress may manifest as physical symptoms due to the psychosomatic relationship between mental and physical health. This emphasizes the necessity of mental health support networks to lessen nurses’ physical and mental health burden. However, the current findings may indicate that factors other than depression contribute to nurses’ physical health outcomes. Zhao et al. [39] emphasized the influence of workplace-related factors, particularly interpersonal conflict and negative emotions at work, on nurses’ psychological well-being. This suggests that occupational and personal factors may play a substantial role in explaining physical health outcomes beyond depression alone.

A modest positive correlation was observed between toxic leadership and intrinsic motivation at the bivariate level. However, this relationship was not supported in the structural model, suggesting that the observed association may reflect the influence of other contextual, organizational, or individual factors not captured in the present model. This finding indicates that intrinsic motivation may operate independently of toxic leadership experiences among mental health nurses. In contrast, previous research has generally suggested that toxic leadership undermines employees’ motivation by creating a hostile work environment, reducing psychological safety, and weakening employees’ sense of autonomy and engagement [40]. The discrepancy between the present findings and previous studies may be attributed to the unique characteristics of mental health nursing settings, where professional commitment, personal resilience, and patient-centered values may help sustain intrinsic motivation despite exposure to challenging leadership behaviors.

The positive correlations between toxic leadership, psychological distress, and somatic symptoms align with prior studies indicating that toxic leadership behaviors, such as bullying and manipulation, have been associated with higher stress levels and adverse health outcomes [41]. Toxic leaders create environments that erode employees’ well-being, and were associated with burnout, anxiety, and physical symptoms.

Interestingly, there was no significant correlation between toxic leadership and OCB. This suggests that toxic leadership may not directly be associated with employees’ discretionary efforts to support their organization. This conclusion contrasts with research indicating that employees’ perceptions of fairness and organizational support are shaped by leadership quality, which has a considerable impact on OCB [42]. The impact of toxic leadership on OCB in this setting may be mitigated by other mediating elements, such as peer connections or organizational culture, which could account for this disparity.

Similarly, the absence of significant relationships between OCB and both psychological distress and somatic symptoms indicates that OCB is not directly affected by individual well-being in the studied population. This contradicts the findings by Organ [43], who suggested that positive mental and physical states often promote proactive organizational behavior. It is possible that in high-stress environments like healthcare, OCB is influenced more by professional norms and external expectations than by individual health conditions.

Theoretical arguments on toxic leadership draw attention to the negative consequences of this leadership style on both organizational and individual levels. Our study findings highlight the complex interplay between toxic leadership, intrinsic motivation, psychological distress, somatic symptoms, and organizational citizenship.

### 4.1. Toxic Leadership and Well-Being Outcomes

The significant direct relationship between toxic leadership and psychological distress supports existing literature that identifies toxic leadership as a significant stressor in organizational settings [44]. It is well-established that toxic leadership practices, like animosity, micromanagement, and a lack of support, make employees’ psychological suffering worse [45]. The biopsychosocial paradigm, which links mental health conditions to physical symptoms, is consistent with the observed positive association between psychological distress and somatic symptoms [46]. These findings further highlight the close association between toxic leadership, psychological distress, and somatic symptoms among mental health nurses.

For instance, a study by Labrague [47] found that toxic leadership significantly increases psychological distress and depressive symptoms among nurses, primarily due to heightened workplace stress and emotional exhaustion. Their findings align with the argument that a hostile work environment created by toxic leaders exacerbates mental health issues, ultimately leading to higher levels of psychological distress. Conversely, Wu et al. [48] suggested that the effects of toxic leadership on nurses’ psychological well-being may be moderated by resilience and perceived organizational support. Their findings indicate that nurses with high resilience or strong institutional backing may not experience severe depressive symptoms despite working under toxic leadership.

### 4.2. Intrinsic Motivation and Organizational Citizenship Behavior

The positive association between intrinsic motivation and organizational citizenship behavior (OCB) repeats the self-determination theory, which posits that intrinsically motivated employees are more likely to engage in discretionary behaviors that benefit the organization [49]. This relationship highlights the importance of fostering intrinsic motivation to enhance workplace harmony and collaboration. Our study is consistent with other studies, many of which support the positive influence of intrinsic motivation on employee discretionary behaviors. For example, Kim et al. [50] found that intrinsically motivated employees are more likely to engage in organizational citizenship behavior because they find personal satisfaction and meaning in their work.

In contrast, a study by Lee et al. [51] suggested that external factors, such as organizational culture and leadership style, may condition the effect of intrinsic motivation on organizational citizenship behavior. Their findings suggested that in highly bureaucratic or authoritarian environments, intrinsically motivated employees may be discouraged from exhibiting organizational citizenship behavior. This paradox suggests that although intrinsic motivation has been associated with higher organizational citizenship behavior, this association may depend on contextual and structural conditions within the organization.

### 4.3. The Role of Intrinsic Motivation in Employee Well-Being Outcomes

Contrary to the proposed hypothesis, the structural model did not provide sufficient evidence to support the mediating role of intrinsic motivation in the relationships between toxic leadership and psychological distress or somatic symptoms among mental health nurses. Although intrinsic motivation was positively associated with organizational citizenship behavior, its direct effects on psychological distress and somatic symptoms were not statistically significant. Consistent with these findings, although the structural model identified a significant indirect association between toxic leadership and somatic symptoms, the structural paths involving intrinsic motivation were not statistically significant. Therefore, this indirect association should not be interpreted as evidence supporting the hypothesized mediating role of intrinsic motivation.

Previous research has explored the complex relationships between toxic leadership, intrinsic motivation, and employee well-being outcomes among healthcare professionals. For example, Koç et al. [26] reported that intrinsic motivation moderated the relationship between toxic leadership and emotional exhaustion, suggesting that higher intrinsic motivation may buffer the negative effects of toxic leadership on employees’ emotional well-being. In contrast, Houkes et al. [52] found that intrinsic motivation was not a significant predictor of emotional exhaustion among nurses, indicating that other organizational and psychological factors may exert a stronger influence on nurses’ well-being.

The present findings are more consistent with the latter perspective, suggesting that intrinsic motivation alone may be insufficient to explain the associations between toxic leadership on psychological distress and somatic symptoms. Mental health nurses are exposed to unique occupational stressors that may directly influence their psychological and physical well-being regardless of their motivational levels. Therefore, additional mechanisms such as workplace support, resilience, coping strategies, and organizational climate should be explored in future studies to better understand how toxic leadership affects employee well-being in mental health settings.

## 5. Conclusions

The present study highlights the significant associations between toxic leadership and psychological distress, as well as between psychological distress and somatic symptoms, among mental health nurses. The findings demonstrated that toxic leadership was positively associated with psychological distress, which in turn was strongly associated with somatic symptoms. These results emphasize the importance of addressing destructive leadership behaviors in mental health settings to protect nurses’ psychological and physical well-being.

The study also revealed that intrinsic motivation was positively associated with organizational citizenship behavior, indicating that intrinsically motivated nurses are more likely to engage in positive discretionary behaviors that support their organizations. However, the hypothesized mediating role of intrinsic motivation in the relationships between toxic leadership and psychological distress or somatic symptoms was not supported by the structural model.

Based on these findings, healthcare organizations should implement leadership development programs that promote supportive leadership practices, emotional intelligence, effective communication, and psychological safety. In addition, strategies aimed at enhancing nurses’ intrinsic motivation, such as professional development opportunities, recognition programs, autonomy in practice, and participative decision-making, may contribute to strengthening organizational citizenship behavior and improving workplace engagement.

Future research should examine additional psychological and organizational mechanisms, including resilience, coping strategies, social support, and organizational climate, that may explain how toxic leadership influences employee well-being among mental health nurses.

### 5.1. Strengths and Limitations of the Study

This study provides valuable insights into the impact of toxic leadership on the well-being and workplace behavior of mental health nurses, particularly its associations with psychological distress, somatic symptoms, intrinsic motivation, and organizational citizenship behavior (OCB). A key strength of this study is its comprehensive approach, which simultaneously examined multiple psychological and organizational variables within a single structural model. This approach contributes to a broader understanding of how leadership behaviors may influence nurses’ psychological well-being and workplace outcomes. Additionally, the findings offer practical implications for healthcare administrators by highlighting the importance of supportive leadership practices in promoting employee well-being and professional engagement.

One of the key strengths of this study is its focus on mental health nurses, a group frequently exposed to high-stress environments due to the emotional and psychological demands of psychiatric care. By examining toxic leadership within psychiatric healthcare settings, this study underscores the critical role of leadership in shaping nurses’ psychological well-being and workplace behaviors. The findings contribute to a deeper understanding of leadership dynamics in mental health institutions and provide targeted recommendations for improving workplace culture, staff retention, and quality of patient care.

Despite the significance of the study findings, several limitations should be acknowledged. First, the study was conducted among 117 mental health nurses from a single psychiatric hospital in Egypt, which may limit the generalizability of the findings to other healthcare settings or countries. Second, the use of self-administered questionnaires may have introduced response bias. Third, the cross-sectional design precludes causal inferences regarding the relationships among the study variables.

Nevertheless, the study focused on a specialized and relatively homogeneous population of mental health nurses working in psychiatric settings. This targeted sample provides valuable insights into the relationships among toxic leadership, intrinsic motivation, psychological distress, somatic symptoms, and organizational citizenship behavior within this unique professional context. Future multicenter studies with larger samples and longitudinal designs are recommended to further validate and extend the present findings.

### 5.2. Future Directions

To address these limitations, future research should employ longitudinal and multicenter studies involving larger and more diverse samples across different healthcare institutions and countries. Such studies would strengthen the generalizability of the findings and provide a better understanding of the long-term effects of toxic leadership on nurses’ psychological well-being and workplace outcomes. Future studies may also benefit from incorporating mixed-method approaches, including qualitative interviews and observational methods, to gain deeper insights into the mechanisms through which toxic leadership affects nurses.

In addition, future research should examine other potential mediating and moderating factors, such as resilience, coping strategies, workplace support, organizational climate, and team dynamics, to better understand the pathways through which toxic leadership influences employee well-being and organizational behavior.

### 5.3. Relevance to Clinical Practice

Addressing toxic leadership is essential for creating a healthy and supportive work environment for mental health nurses. Healthcare administrators and nurse managers should prioritize leadership development programs that promote emotional intelligence, effective communication, psychological safety, and supportive leadership practices. Such initiatives may help reduce workplace stress and enhance nurses’ psychological well-being.

The findings of this study demonstrated a significant association between toxic leadership and psychological distress, as well as a strong relationship between psychological distress and somatic symptoms. These findings highlight the importance of implementing mental health support services, stress-management interventions, and regular well-being assessments for nurses working in psychiatric settings.

Furthermore, intrinsic motivation was positively associated with organizational citizenship behavior, suggesting that healthcare organizations should foster professional autonomy, competence development, recognition, and opportunities for professional growth. Creating a supportive and collaborative workplace culture may encourage positive discretionary behaviors that contribute to organizational effectiveness and improved patient care outcomes.

Healthcare institutions should also establish transparent policies, leadership accountability mechanisms, and employee feedback systems to identify and address toxic leadership behaviors. Such measures can contribute to healthier work environments, improved staff retention, and better outcomes for both nurses and patients.

## Figures and Tables

**Table 1:** Mental health nurses’ demographics and variances in the study variables (n = 117).

Characteristic	Category	No. (%)	Toxic Leadership		Intrinsic Motivation		Organizational Citizenship Behavior		Psychological Distress		Somatic Symptoms	
			M (SD)	*t*/f (*p*)	M (SD)	*t*/f (*p*)	M (SD)	*t*/f (*p*)	M (SD)	*t*/f (*p*)	M (SD)	*t*/f (*p*)
Gender	Male	31 (26.5)	2.84 (0.847)	1.31 (0.470)	3.26 (0.605)	−0.71 (0.421)	4.06 (0.667)	0.29 (0.638)	2.70 (1.10)	0.64 (0.506)	2.35 (1.1)	0.41 (0.565)
	Female	86 (73.5)	2.76 (0.930)		3.39 (0.613)		3.94 (0.715)		2.46 (1.09)		2.08 (1.12)	
Age	20–25	38 (32.5)	2.51 (0.880)	4.426 * (0.014)	3.16 (0.589)	4.452 * (0.014)	4.05 (0.757)	1.313 (0.273)	2.10 (1.02)	0.433 (0.650)	2.35 (0.999)	1.316 (0.272)
	25–30	32 (27.4)	2.70 (0.700)		3.32 (0.438)		3.80 (0.649)		2.01 (0.884)		2.39 (0.896)	
	30–35	47 (40.2)	3.05 (0.900)		3.54 (0.686)		4.03 (0.686)		2.24 (1.28)		2.70 (1.21)	
Marital state	Single	62 (53.0)	2.69 (0.921)	−0.890 (0.308)	3.37 (0.558)	12.29 (0.149)	4.04 (0.649)	0.967 (0.315)	2.37 (1.04)	−0.164 (0.636)	2.10 (1.14)	−1.96 (0.453)
	Married	55 (47.0)	2.88 (0.797)		3.35 (0.674)		3.90 (0.754)		2.65 (1.09)		2.16 (1.06)	
Department	Mental Health	99 (84.6)	2.75 (0.87)	−4.43 (0.670)	3.36 (0.59)	0.42 (0.361)	4.03 (0.68)	1.43 (0.257)	2.45 (1.10)	−2.64 (0.433)	2.08 (1.12)	−3.47 (0.448)
	Addiction	18 (15.4)	2.93 (0.83)		3.31 (0.73)		3.70 (0.74)		2.91 (0.97)		2.57 (1.05)	
Years of experience	Less Than 5 Years	105 (89.7)	2.7 (0.82)	0.98 (0.377)	3.33 (0.61)	0.86 (0.425)	3.95 (0.70)	1.34 (0.264)	2.51 (1.07)	0.29 (0.746)	2.12 (1.08)	0.812 (0.446)
	From 5 To Less Than 10	10 (8.5)	3.09 (1.07)		3.60 (0.58)		4.29 (0.52)		2.55 (1.24)		2.29 (1.44)	
	More Than 10	2 (1.7)	3.20 (1.98)		3.28 (0.75)		3.60 (1.13)		3.11 (1.97)		3.09 (1.93)	

M = Mean. SD = Standard deviation. *t* = *t*-test for the independent group. * *p* is significant at the 0.05 level (two-tailed). *p* is significant at the 0.01 level (two-tailed).

**Table 2:** Analyzing the correlation coefficients among the study variables (n = 117).

Studied Variables		Mean (SD)	Toxic Leadership	Intrinsic Motivation	Organizational Citizenship Behavior	Psychological Distress	Somatic Symptoms
Toxic Leadership	R.	2.78 (0.86)	1	0.276 **	−0.004	0.454 **	0.427 **
	Sig			0.003	0.963	0.000	0.000
Intrinsic Motivation	R.	3.35 (0.61)	0.276 **	1	0.453 **	0.279 **	0.278 **
	Sig		0.003		0.000	0.002	0.002
Organizational Citizenship Behavior	R.	3.97 (0.70)	−0.004	0.453 **	1	−0.028	0.027
	Sig		0.963	0.000		0.761	0.774
Psychological Distress	R.	2.52 (1.09)	0.454 **	0.279 **	−0.028	1	0.947 **
	Sig		0.000	0.002	0.761		0.000
Somatic symptoms	R.	2.15 (1.12)	0.427 **	0.278 **	0.027	0.947 **	1
	Sig		0.000	0.002	0.774	0.000	

R. = Pearson correlation. ** Correlation is significant at the 0.01 level (two-tailed).

**Table 3:** Direct and indirect associations obtained from the structural equation model (n = 117).

Effects	*β*	*p*	BC 95% CI Lower/Upper
Direct effects			
Toxic Leadership and Intrinsic Motivation	−0.12	0.18	−0.39/0.04
Toxic Leadership and Psychological Distress	0.46	0.00	−0.22/0.69
Toxic Leadership and Organizational Citizenship Behavior	−0.01	0.68	−0.06/0.06
Intrinsic Motivation and Psychological Distress	−0.03	0.79	−0.03/0.19
Intrinsic Motivation and Organizational Citizenship	0.31	0.00	0.18/0.50
Intrinsic Motivation and Somatic Symptoms	0.02	0.69	−0.09/0.11
Psychological Distress and Somatic symptoms	0.99	0.00	0.82/1.25
Indirect effects			
Toxic Leadership and Somatic Symptoms	0.46	0.00	0.26/0.68
Toxic Leadership and Organizational Citizenship	−0.04	0.23	−0.12/0.02
Toxic Leadership and Psychological Distress	0.00	0.78	−0.03/0.12
Intrinsic Motivation and Somatic Symptoms	−0.03	0.74	−0.33/0.16

(*p*) Significant level < 0.05. (*p*) Significant level < 0.01.

**Table 4:** The fit indices for structural equation modeling analysis (n = 117).

Indices	χ^2^/df	*p*	RMSEA	NFI	RFI	AGFI	TLI
Model	3.491	0.06	0.047	0.98	0.97	0.92	0.97

Abbreviations: χ^2^/df = Chi-square divided/degrees of freedom; RMSEA = Root mean square error of approximation; NFI = Normed fit index; RFI = Relative fit index; AGFI = Adjusted goodness of fit index; TLI = Tucker–Lewis index.

## Data Availability

The data presented in this study are available upon request from the corresponding author due to ethical and privacy restrictions related to sensitive participant information.
